# Effects of Exposure to Community Violence and Family Violence on School Functioning Problems among Urban Youth: The Potential Mediating Role of Posttraumatic Stress Symptoms

**DOI:** 10.3389/fpubh.2014.00008

**Published:** 2014-02-07

**Authors:** Tia M. McGill, Shannon R. Self-Brown, Betty S. Lai, Melissa Cowart-Osborne, Ashwini Tiwari, Monique LeBlanc, Mary Lou Kelley

**Affiliations:** ^1^School of Public Health, Georgia State University, Atlanta, GA, USA; ^2^Department of Psychology, Southeastern Louisiana University, Hammond, LA, USA; ^3^Department of Psychology, Louisiana State University, Baton Rouge, LA, USA

**Keywords:** community violence exposure, family violence exposure, adolescent posttraumatic stress symptoms, mediator, school adjustment

## Abstract

Adolescents who are exposed to violence during childhood are at an increased risk for developing posttraumatic stress (PTS) symptoms. The literature suggests that violence exposure might also have negative effects on school functioning, and that PTS might serve as a potential mediator in this association. The purpose of the current study was to replicate and extend prior research by examining PTS symptoms as a mediator of the relationship between two types of violence exposure and school functioning problems among adolescent youth from an urban setting. Participants included a sample of 121 junior high and high school students (*M* = 15 years; range = 13–16 years; 60 males, 61 females) within high-crime neighborhoods. Consistent with our hypotheses, community violence and family violence were associated with PTS symptoms and school functioning problems. Our data suggest that community and family violence were indirectly related to school functioning problems through PTS symptoms. Findings from this study demonstrate that PTS symptoms potentially mediate the relationship between violence exposure and school functioning problems across two settings (community and home). Future research should further examine protective factors that can prevent youth violence exposure as well as negative outcomes related to violence.

## Introduction

Adolescent violence exposure and victimization, in the community and home, are a major public health concern in the United States. Among youth aged 14–17 years, 70% reported lifetime victimization of physical assault and almost 40% reported being victims of assault within the past year (1). During 2010, approximately 1.2 million children aged 12–17 years lived in a household in which a violent crime against a youth occurred during that year (2, 3). Adolescents living in urban communities, marked by poverty, crime, and drug-related activities, are often at increased risk for violence exposure and victimization, including homicides, assaults, and physical altercations (4–7). Given the high rates of direct and indirect violence exposure among urban adolescents, there is significant concern regarding the adverse impact of exposure to violence on youth functioning and development.

A vast body of work exists regarding the harmful mental health effects of pervasive youth violence exposure with a significant focus on posttraumatic stress (PTS) symptomatology as an outcome (7–11). For instance, community violence studies indicate that approximately 3–19% of youth have been found to be at risk for developing PTS (12–14). Additionally, in family violence studies, approximately 13–50% of exposed youth have been found to be at risk for PTS (15–20).

In addition to PTS symptoms, violence-exposed youth living in urban communities are at significantly higher risk for developing school problems including lower grade point averages (21–25), decreased standardized test scores in reading and math (24, 25), and poor school attendance (21). Although there is emerging evidence documenting a robust association between youth violence exposure and poor school outcomes, some violence-exposed youth maintain high levels of adaptive school functioning and overall positive trajectories (26). Thus, it appears that there are mechanisms impacting the youth violence-school outcome relation that mitigate the conditions under which violence exposure leads to adverse outcomes (27). The current study will focus on adolescent PTS symptoms as a potential mediator between youth violence exposure and school functioning problems.

To date, one known study has examined the association between exposure to community violence, PTS symptoms, and school outcomes. Specifically, Mathews et al. (28) examined the role of youth PTS symptoms as a mediator between community violence, academic performance (student grade point average, standardized test percentile ranking), and school attendance in a sample of 47 pre-adolescent youth (aged 10–13 years) living in an urban environment. PTS symptoms emerged as a mediator between community violence and academic performance, but not for the relation between community violence and school attendance. The current study proposes to replicate the findings of Mathews and colleagues by examining PTS symptoms as a mediator in the relation between community violence and school functioning problems, defined as youth attitude toward school, attitude toward teachers, and sensation seeking, in a slightly older, adolescent sample of urban youth (aged 13–16 years). While developmental differences do emerge between pre-adolescence and adolescence in regard to the variables of interest in this study, a mediating relation between violence exposure, PTS, and school functioning is still anticipated. The role of child characteristics, such as parent education level, adolescent gender, and age, will also be considered in the examination of the interplay between violence, PTS symptoms, and school functioning problems, as past research has supported that lower levels of parental educational attainment is a risk factor for violence exposure (29–32); girls and younger children are more likely to express PTS as a result of violence exposure (33–36) and male gender has been associated with poorer school functioning problems (37, 38).

The environment in which violence is experienced can make significant differences for youth trajectories and can be important for treatment planning (39). Therefore, it is critical to measure multiple types of violence exposure in youth, as well as the related outcomes. The current study will expand upon the Mathews and colleagues findings by also exploring the relation between youth family violence exposure and school functioning problems using cross-sectional analyses. No known research to date has examined the relations between family violence exposure, PTS symptoms, and school functioning problems. Given that PTS symptoms among youth are associated with increased school functioning problems such as slower and less effective learning (40); poor attention (41); and poor overall short-term academic functioning (42); it was hypothesized that youth community and family violence exposure would be associated with school functioning and that youth PTS symptoms would serve as a potential mediator of these associations.

## Materials and Methods

### Participants

This study was completed using secondary data analyses from a convenience sample of junior high and high school students and their parent/guardians (*n* = 138) recruited from schools located in high-crime neighborhoods within a mid-sized southern city. Participant characteristics included 60 males and 61 females with a mean age of 15 (ranging from 13 to 16 years). Due to unusable, incomplete, or invalid data, 17 respondents of the parent–child dyads were excluded from the study, yielding 121 participant pairs included in the analyses. The study sample was comprised of mostly minority students and parents (99%) with African-American students accounting for the largest ethnic group (97%). The sample was predominantly of low socioeconomic status, with 71% reporting an annual income of less than $20,000. Additionally, the free and reduced lunch rates across all schools attended ranged from 74 to 86%. Schools were located in areas, which had crime rates higher than the national average for murders, robberies, aggravated assaults, burglaries, and theft (43).

### Materials

A full listing of measures used in the original study can be found in the Self-Brown et al. manuscript (44). Select sources of data were analyzed for the purposes of this study.

#### Demographic questionnaire

Participants provided information on child age, grade, gender, and race/ethnicity, as well as parent age, marital status, education level, occupation, and income level on this one-page survey.

#### Measures of violence

The Screen for Adolescent Violence Exposure [SAVE; (45)] was completed by each student to assess levels of community and family violence. The SAVE is an adolescent self-report scale, which assesses the frequency of violence exposure in settings relevant to adolescent adjustment (School, Neighborhood, and Home). The SAVE consists of 32 items, which are administered in a five-point Likert format. Three factors have been identified for each setting scale: Traumatic Violence, Indirect Violence, and Interpersonal Aggression. The SAVE has been found to successfully classify low- and high-violence groups, and demonstrate good internal consistency, test–retest reliability, and validity. The SAVE has obtained significant correlations with both independent violence data and theoretically related constructs (45). The School and Neighborhood subscales were used as a measure of adolescent community violence exposure, while the home subscales were used as a measure of adolescent family violence exposure.

#### PTS symptoms

The *Trauma Symptom Checklist for Children [TSCC; (46)*] was completed by each student to assess PTS symptoms. The TSCC is a self-report measure of PTS and related psychological symptomatology in children aged 8–16 years. The full version of the TSCC consists of 54 items that yield validity scales (Under Response and Hyper Response) and clinical scales (Anxiety, Depression, Anger, PTS, Dissociation, and Sexual Concerns). The TSCC has demonstrated high internal consistency, as well as construct, convergent, and discriminant validily (46). The PTS clinical scale was utilized as a measure of adolescent PTS symptomatology in this study.

#### School functioning problems

The Behavior Assessment System for Children II-Self-Report of Personality (BASC-2 SRP) was completed by each student to assess school functioning problems (47). The *BASC-2 SRP* is a 186-item, self-report instrument designed to measure an individual’s risk level of their self-perceived personality, affect, and attitudes (47). The BASC-2 SRP demonstrates good psychometric properties including good internal consistency reliability (0.85–0.96) and good adjusted test–retest reliability (0.75–0.80). The BASC-2 SRP measures five domains including: Inattention/Hyperactivity, Internalizing Problems, Personal Adjustment, School Problems, and the Emotional Symptoms Index. For the purpose of this study, the composite score for School Problems (Attitude to School, Attitude to Teachers, and Sensation Seeking) was used as an outcome variable. Scores were interpreted such that higher composite scores indicated higher risk for maladjustment.

### Procedure

Adolescents in grades 7–11 were recruited during lunchtime at local urban junior high and high schools. Those interested in participating in the study were provided with a brief explanation of the study as well as verbal and written instructions delineating the procedures and time commitment involved. Adolescents who agreed to participate were given packets containing study measures. Verbal and written instructions were provided, which explained the adolescent that he/she was responsible for taking the packet home, reading over and signing the assent form, completing the adolescent self-report portions of the packet which included the SAVE and the TSCC, having the parent/guardian read over and sign the consent form, and complete the parent portion of the packet. Adolescents were required to return the packet within 1 week. Parent and adolescent responses were anonymous and packets were coded to match parent and adolescent data. Following completion of the questionnaires, the adolescents were debriefed regarding the purposes of the study, at which time they were given the opportunity to ask questions regarding the study and the measures they completed. Referral cards for psychological services were provided to all participants. For their time, participants were paid $5. Following data collection, 10%, or 13 parents, were contacted to ensure that the adolescents had not falsified parental data. All parents contacted and indicated that they had signed the consent form and completed the parent questionnaires included in the study materials.

### Statistical analyses

Bivariate correlation analyses were conducted to examine the relationships between study variables: parent education level as well as adolescent gender, age, PTS symptoms, family violence exposure, community violence exposure, and school functioning problems.

Next, we utilized structural equation models (using Mplus, version 7.11) as a suitable method to test study hypotheses (48). Model fit was evaluated based on the following criteria: non-significant χ^2^; CFI > 0.95; RMSEA < 0.06; and SRMR < 0.10 (49, 50). To test PTS as an intervening variable, we examined the significance of the joint effects between: (i) family violence or community violence to PTS symptoms, and (ii) PTS symptoms to school functioning problems.

## Results

### Bivariate correlations

Bivariate correlation analyses were conducted for all study variables (see Table 1). For demographic variables, parent education level correlated significantly with family violence exposure such that parents who reported higher levels of education had lower levels of family violence. Student gender was positively and significantly correlated with adolescent PTS symptoms indicating that females were rated as experiencing higher levels of PTS symptomatology. Based on significant correlations, gender was included as a covariate of PTS symptoms in study analyses, and parent education level was included as a covariate for family violence exposure in study analyses. In addition, given evidence that gender is associated with school functioning problems, and that younger age is associated with PTS symptoms, these variables were included as covariates.

**Table 1:** **Correlation matrix for study variables**.

Variable	1	2	3	4	5	6	7
1. Parent education level	–	0.06	0.05	−0.16	−0.19*	−0.09	0.041
2. Adolescent gender		–	0.13	0.24**	0.10	0.09	−0.06
3. Adolescent age			–	−0.08	0.03	0.01	0.06
4. Adolescent PTS symptoms				–	0.49**	0.42**	0.45**
5. Adolescent community violence exposure					–	0.77**	0.33**
6. Adolescent family violence exposure						–	0.41**
7. Adolescent school functioning							–

*p < 0.05, **p < 0.01.

For main study variables, community violence exposure was positively and significantly correlated with family violence exposure and school functioning problems. The intervening variable under study, adolescent PTS symptoms, was significantly and positively linked to community and family violence exposure, as well as school functioning problems. None of the demographic variables of interest were significantly correlated with school functioning problems. The moderate correlation coefficient between adolescent PTS symptoms, community violence exposure, family violence exposure, and school functioning variables provided some confidence in the analysis (51).

### PTS symptoms as a potential mediator

#### Community violence exposure

**Figure 1:**
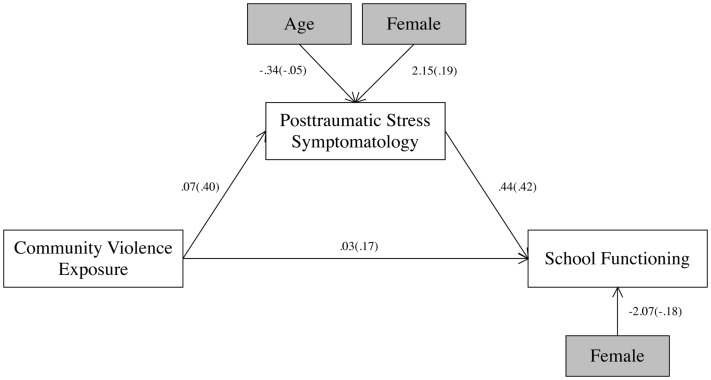
**Community violence, PTS symptoms, and school functioning problems**. All paths were significant (*p* < 0.05). Unstandardized path coefficients are listed, followed by standardized path coefficients in parentheses.

Associations between community violence exposure, PTS symptoms, and school functioning problems were examined with structural equation modeling (see Figure 1). This model fit the data well, χ^2^(1) = 0.78, *p* = 0.38, CFI = 1.00, RMSEA = < 0.001, SRMR = 0.02, and this model explained 26% of the variance in school functioning problems. In this model, school functioning problems were associated with male gender, *b* = −2.07, SE = 0.93, *p* = 0.03. PTS symptoms were significantly associated with female gender, *b* = 2.15, SE = 0.91, *p* = 0.02, but not age, *b* = −0.34, SE = 0.51, *p* = 0.50. Community violence was directly related to school functioning problems, *b* = 0.03, SE = 0.02, *p* = 0.04. PTS symptoms were also directly related to school functioning problems, *b* = 0.44, SE = 0.09, *p* < 0.001. The model path for the indirect effect of community violence through PTS symptoms to school functioning problems was significant, *b* = 0.03, SE = 0.01, *p* = 0.001 (see Table 2). This indirect effect is consistent with mediation, indicating that PTS symptoms are a potential mediator of the relationship between community violence and school functioning problems.

**Table 2:** **Model fitting results**.

Model fit	Community violence		Family violence	
	χ^2^(1) = 0.78, *p* = 0.38, CFI = 1.00, RMSEA = < 0.001, SRMR = 0.02		χ^2^(5) = 4.31, *p* = 0.51, CFI = 1.00, RMSEA = < 0.001, SRMR = 0.04	
		
Explanatory variables	Unstandardized parameter estimates	Standard error	Unstandardized parameter estimates	Standard error
**DEMOGRAPHIC FACTORS**				
Age to PTS symptoms	−0.34	0.51	−0.43	0.48
Female gender to PTS symptoms	2.15	0.91	2.00	0.87
Female gender to school functioning	−2.07	0.93	−2.04	0.92
Parent education to violence	N/A	N/A	−3.52	1.64
**VIOLENCE FACTORS**				
Violence to school functioning	0.03	0.02	0.06	0.02
Violence to PTS symptoms	0.07	0.01	0.11	0.02
**PTS SYMPTOMS**				
PTS symptoms to school functioning	0.44	0.09	0.39	0.09

#### Family violence exposure

**Figure 2:**
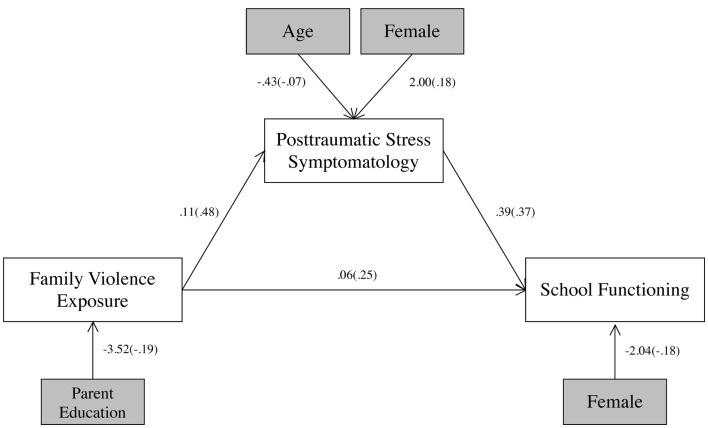
**Family violence, PTS, and school functioning problems**. All paths were significant (*p* < 0.05). Unstandardized path coefficients are listed, followed by standardized path coefficients in parentheses.

Associations between family violence exposure, PTS symptoms, and school functioning problems were examined with structural equation modeling (see Figure 2). This model fit the data well, χ^2^(5) = 4.31, *p* = 0.51, CFI = 1.00, RMSEA = < 0.001, SRMR = 0.04, and this model explained 29% of the variance in school functioning problems. In this model, school functioning problems were associated with male gender, *b* = −2.04, SE = 0.92, *p* = 0.03. PTS symptoms were significantly associated with female gender, *b* = 2.00, SE = 0.87, *p* = 0.02, but not age, *b* = −0.43, SE = 0.48, *p* = 0.37. Further, family violence was negatively associated with parent education, *b* = −3.52, SE = 1.64, *p* = 0.03. Family violence was directly related to school functioning problems, *b* = 0.06, SE = 0.02, *p* = 0.01. PTS symptoms were also directly related to school functioning problems, *b* = 0.39, SE = 0.09, *p* < 0.001. The model path (see Table 2) for the indirect effect of family violence through PTS symptoms to school functioning problems was significant, *b* = 0.04, SE = 0.01, *p* = 0.001. This indirect effect is consistent with mediation, indicating that PTS symptoms are a potential mediator of the relationship between family violence and school functioning problems.

## Discussion

The present study explored the association between adolescent violence exposure and school functioning problems with the purpose of examining whether student PTS symptomatology was a potential mediator of this association for a sample of 121 urban youth. A recent study by Mathews and colleagues (28) found PTS to be a mediator in the association of community violence exposure and poor school functioning for pre-adolescent urban youth aged 10–13 years. The current study attempted to replicate these findings with youth aged 13–16 years, as well as extend prior research by exploring the unique and cumulative effects of family violence for adolescent urban youth. Exploring both community and family violence exposure allowed for a better understanding of how setting impacts these associations. Hypotheses for this study were fully supported.

Consistent with existing research, results of this study revealed a significant association between community violence and negative school functioning (22, 23, 25, 28), which was defined as a poor attitude toward school and teachers and heightened sensation seeking. Furthermore, findings revealed that PTS symptomatology is a primary mechanism through which exposure to community violence impacts school functioning. That is, symptoms of PTS emerged as a potential mediator in the relation between community violence exposure and school functioning problems among this study sample, replicating the findings by Mathews et al. (28) in an urban sample of older youth. Urban youth living in communities marked by violence are at increased risk for PTS symptomatology. These symptoms include re-experiencing and hyperarousal, which might both lead to distraction from full engagement in school activities and make it difficult to complete school work in an effective manner, further demonstrating that PTS symptoms impact student learning. Symptoms of avoidance might also lead students to actively avoid engaging in relationships with peers and teachers, and thus have a more negative attitude toward school from an academic and social perspective.

In terms of family violence, study results indicated that youth exposed to family violence have poorer school functioning, and that PTS might be a primary mechanism involved in this association. These findings expand upon several previous investigations which have described links between violent home environments, marked by the presence of domestic violence and parent–child abuse, with lower youth academic achievement (52–55) by also identifying an important mediator involved in this association. Violence in the home is more proximal to youth than community violence and past research has indicated that violence exposure in this setting is more strongly associated with youth PTS symptoms than community violence (44, 56). As a potential mediator of the family violence-school functioning problems association, PTS likely impacts school functioning as described above, through symptoms such as intrusive thoughts, increased arousal, avoidance, and irritability, which negatively impact students’ ability and motivation to positively engage in the school environment.

In this study sample, several demographic characteristics were examined to explore how they impact the relation between community violence, family violence, PTS, and school functioning. In both models, female respondents were more likely than male respondents to report PTS symptoms. The predominance of female cases is a frequent and consistent finding in the study of youth PTSD symptoms (34, 57), possibly as a result of the types of violent events to which the youth are exposed, or due to genetic and/or social factors (58). Further, across both models, males were more likely to have poor school functioning than females, which is also a consistent finding in past research (37, 38). While gender was related to both school functioning and PTS symptoms, age did not emerge as a significant influential factor in the relationship between exposure to community or family violence and the presence of PTS or school problems. It is likely that the restrictive age limit among the current study sample warrants further investigation into how age and other demographic factors influence the relation between exposure to violence, the development of PTS, and school functioning, as age has also been found to impact adolescent sensation seeking, attitude toward school, and attitude toward teachers. Lastly, a lower level of parent education was associated with higher levels of family violence exposure, which is a consistent finding in the area of family violence research (59–61).

### Limitations

Findings of this study should be regarded in the context of this study and interpreted cautiously due to several study limitations. First, the correlational nature of this study did not allow for causal associations to be established. Second, the use of cross-sectional data did not allow for assessment of the impact of time in the process of experiencing exposure to community and family violence and experiencing poor school functioning, which would require longitudinal data. Third, the sample was a convenience sample from a secondary data set, recruited from schools in a high violence, restricted area of a mid-sized southern city. This introduced selection bias has increased the risk of confounding variables, thereby decreasing generalizability of study findings, since results might be uniquely influenced by locale. Fourth, this study did not differentiate between youth victimization versus witnessing of community or family violence. Fifth, there were several limitations to measurement including: (a) school outcome was limited to a composite scale of self-report items and did not include concrete measures of school outcomes, such as grade point average (GPA) or achievement testing, (b) measurement did not include other forms of student psychopathology that often overlap with PTSD, such as Attention Deficit Hyperactivity Disorder, or Bipolar Disorder, (c) the PTSD measure utilized was based on DSM-IV diagnostic criteria, (d) no observational measures or verbal report of symptomatology were included, and (e) no multi-informant indicators of school outcome from teachers or parents were included. Researchers have argued that exclusive use of self-report raises the possibility of shared method variance and prohibit a comprehensive assessment of child functioning (24, 62). Given the problems inherent in the exclusive use of adolescent self-report, a more valid approach would be to gather data from multiple informants, which would allow for a comprehensive assessment of adolescent functioning and might produce more reliable results across studies (63, 64).

### Future research

Despite limitations, the current study should serve as a guide to develop future research as it provides support for future work regarding the contribution of adolescent PTS to the school functioning problems of urban youth exposed to violence. There are several recommendations for additional research to better understand this relation. There were a number of confounding variables, which were beyond the scope of the current study, that could be statistically related to adolescents’ exposure to family and community violence as well as school functioning and warrant further study. For example, empirical evidence suggests that variables such as: adolescent’s attachment history (65), history of neglect and presence of substance abuse (66) as well as other family stressors such as: financial problems, divorce, and overcrowding (67) are important factors related to violence exposure and school adjustment.

Future research should examine how a diagnosis of posttraumatic stress disorder (PTSD), as compared to a range in symptoms, influences the relation between violence exposure and school functioning problems. Additionally, future research should utilize established and concrete measures of school functioning and school potential, such as intellectual functioning measures, graduation rates, grade point average, and scores on standardized tests of academic performance. Furthermore, evidence suggests that adolescents’ verbal reports differ from their responses on written questionnaires thereby strengthening the rationale for multiple assessments of PTS symptoms (68). Future studies would benefit from including clinical interviews to ensure that verbal responses are consistent with written self-report assessments.

Next steps in this research could also include an examination of protective factors, such as sources of social support, the effects of neighborhood and school programs, and neighborhood cohesion, to increase the overall understanding of what factors lead to resilience in urban youth exposed to violence. Given our results, it would perhaps have been interesting to take into consideration other psychological consequences of exposure to community and family violence such as depression, behavior problems, and substance abuse. Finally, there is a dire need for longitudinal research to validate the cross-sectional findings of this and other studies, and to explore new pathways between exposure to violence and school functioning problems, focusing on youth mental health diagnoses other than PTS.

## Author Contributions

Tia M. McGill formulated the study aims, prepared the manuscript, and carried out analyses. Shannon R. Self-Brown supervised all aspects of the research process, prepared the manuscript, and carried out analyses. Betty S. Lai conducted analyses and edited the manuscript. Ashwini Tiwari conducted literature searches, created and maintained citation database, and edited the manuscript. Melissa Cowart-Osborne conducted literature searches, edited the manuscript, and participated in data analysis. Monique LeBlanc provided statistical consultation and manuscript editing. Mary Lou Kelley was the primary supervisor of data collection, Institutional Review Board approval, and was primarily responsible for the relationship with east Baton Rouge Parish school board that allowed for data collection.

## Conflict of Interest Statement

The authors declare that the research was conducted in the absence of any commercial or financial relationships that could be construed as a potential conflict of interest.
